# Human Chorionic Gonadotropin Beta Subunit Genes *CGB1* and *CGB2* are Transcriptionally Active in Ovarian Cancer

**DOI:** 10.3390/ijms140612650

**Published:** 2013-06-17

**Authors:** Marta Kubiczak, Grzegorz P. Walkowiak, Ewa Nowak-Markwitz, Anna Jankowska

**Affiliations:** 1 Department of Cell Biology, Poznan University of Medical Sciences, Rokietnicka 5D, 60-806 Poznan, Poland; E-Mails: kubiczak@ump.edu.pl (M.K.); walkowiak@ump.edu.pl (G.P.W.); 2 Department of Gynecologic Oncology, Poznan University of Medical Sciences, Polna 33, 60-535 Poznan, Poland; E-Mail: ewamarkwitz@poczta.fm

**Keywords:** human chorionic gonadotropin beta subunit, *CGB* gene expression, *CGB1 CGB2*, ovarian cancer

## Abstract

Human chorionic gonadotropin beta subunit (CGB) is a marker of pregnancy as well as trophoblastic and nontrophoblastic tumors. CGB is encoded by a cluster of six genes, of which type II genes (*CGB3/9*, *5* and *8*) have been shown to be upregulated in relation to type I genes (*CGB6/7*) in both placentas and tumors. Recent studies revealed that *CGB1* and *CGB2*, originally considered as pseudogenes, might also be active, however, the protein products of these genes have not yet been identified. Our study demonstrates the presence of *CGB1* and *CGB2* transcripts in ovarian carcinomas. While *CGB1* and *CGB2* gene activation was not detected in normal ovaries lacking cancerous development, our study demonstrates the presence of *CGB1* and *CGB2* transcripts in 41% of analyzed ovarian cancer cases.

## 1. Introduction

Human chorionic gonadotropin (CG) is a member of the glycoprotein hormone family. All family members are heterodimers composed of two subunits; common alpha and specific beta determining the structural and functional identity of each hormone [1].

CG plays a crucial role in early pregnancy by regulating a series of processes such as: maternal recognition of pregnancy, embryo implantation, placentation, placental angiogenesis, delaying the apoptosis of the *corpus luteum,* and modulation of maternal/fetal immune responses [2].

Expression of human chorionic gonadotropin beta subunit (CGB) is now a recognised phenomenon of 30%–50% of malignant tumors of various origins [3]. The biological function of CGB is not fully understood, but increased serum levels of CGB are associated with a metastatic phenotype of cancer, its resistance to therapy and poor prognosis [4–8]. It is suggested that CGB acts as an autocrine factor affecting tumor growth by inhibiting apoptosis.

The chorionic gonadotropin beta subunit is encoded by six highly homologous genes, numbered from *CGB1* to *CGB9*, which are arranged in tandem and inverted pairs on chromosome 19q13.3. The expression level of individual *CGBs* is not equal [9]. The three so-called type II genes: *CGB3/9*, −*5* and −*8* have been shown to be upregulated in relation to the type I genes, *CGB6/7*, in placenta and some tumors [10–12]. It was demonstrated that the ratio of type II to type I *CGB* gene expression increases in cancers with higher stage and grade and that the ratio is lower in benign than in malignant tissue. This implies that the increase in the expression level of type II genes could be associated with disease progression and differentiation of tumor cells [9].

Recent studies demonstrated that both *CGB1* and *CGB2*, originally believed to be pseudogenes, might be transcriptionally active. Their transcripts differ from each other by only 10 nucleotides but they are highly diverse from the other *CGB* genes. As a result of DNA fragment insertion, *CGB1* and *CGB2* possess a novel 5′UTR sequence and exon 1. Additionally exons 2 and 3 are frameshifted, and the putative proteins of both genes are dissimilar to any known protein [10]. Transcripts of these genes were detected in non-malignant tissue: placenta [10,13], testis [14,15] pituitary [16] as well as cancerous breast tissue [11]. It was recently shown that a high number of alternatively spliced variants was detected in testis and that CGB-derived peptides may arise from the major spliced form [15].

The upstream region of both genes have been shown to contain almost a complete and conserved sequence of CGB-coding gene promoter. In addition *CGB1* and *CGB2* possess a novel, putative proximal promoter fragment, created by a gene-specific insertion, which is predicted to include several regulatory elements essential for CGB expression [17]. The *in silico* recognised transcriptional factor binding sites indicate that *CGB1* and *CGB2* are involved in implantation and placental development, as well as other processes regulated by human chorionic gonadotropin.

Guided by this information, we decided to analyse the activity of *CGB1* and *CGB2* in ovarian cancer tissues in order to compare their expression pattern with normal ovaries lacking cancerous changes and CG-producing placentas. The results of our study demonstrated that *CGB1* and *CGB2* genes are not active in normal ovaries and their activity characterises only tumor tissues (41% of analyzed cases).

## 2. Results and Discussion

Human chorionic gonadotropin beta subunit encoding genes’ expression was evaluated in ovarian cancer tissues, healthy ovaries and term placentas using the qPCR (real-time PCR) method. *CGB* gene activity was evaluated in three separate groups: *CGB1-2*, allowing the detection of *CGB1* and *CGB2* gene activity only; *CGB3-9* comprising *CGB3*, *CGB5*, *CGB6*, *CGB7*, *CGB8*, *CGB9*; and total *CGB*, quantifying all genes encoding the beta subunit of the hormone.

The results of the study demonstrated transcriptional activity of *CGB* genes in examined tissues and showed that their expression pattern varies between studied groups.

### 2.1. Total *CGB* as well as *CGB3-9* Gene Expression Characterized Both Normal Ovaries and Ovarian Cancer

**Figure 1:**
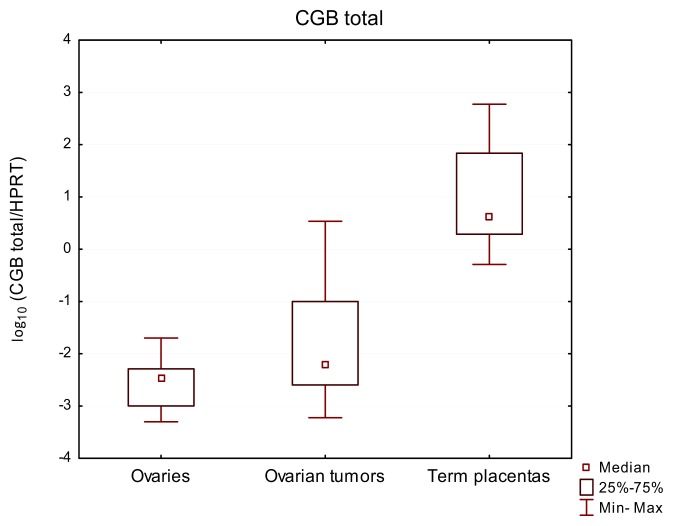
Relative expression of total *CGB* genes in normal ovarian tissues, ovarian carcinomas and term placentas. Results are presented as the logarithm to the base 10.

Bearing in mind the fact of tumor cellular heterogeneity and genetic instability [18], first total expression of *CGB* genes was analyzed, which confirmed their activity in all studied samples. Although gene transcripts were also found in ovaries lacking cancerous changes, the level of their expression was lower than in ovarian cancer tissues and the positive control group of the study—term placentas—showing the highest *CGB* expression level (Figure 1, Table 1). The median value of total *CGB* gene expression equaled 44.3 for placentas, 6.48 × 10^−3^ for ovarian cancer tissues and only 3.51 × 10^−3^ for normal ovaries. The differences between expression of *CGB* genes in ovarian cancer tissue, healthy ovaries and placentas were found to be statistically significant (*p*-value < 0.0001).

**Figure 2:**
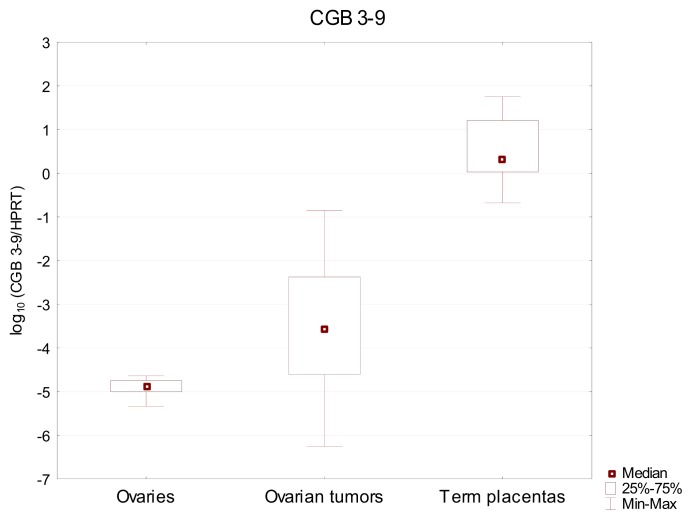
Relative expression of *CGB3-9* genes in normal ovarian tissues, ovarian carcinomas and term placentas. Results are presented as the logarithm to the base 10.

Similar results were observed, when the expression of *CGB3-9* genes was studied. Transcripts of these genes were detected in both ovaries and ovarian cancer, as well as in term placentas. The expression of *CGB3-9* showed greater differences between analyzed tissues and allowed for better dissemination of the studied group. Also this time the highest *CGB* expression level—median value 22.1—was found in the control group of term placentas. In comparison to placenta, the activity of *CGB3-9* in ovarian cancer (2.65 × 10^−4^) and normal ovary (1.31 × 10^−5^) was found to be 4 to 5 orders of magnitude lower and varied greatly (Figure 2, Table 1). These differences were confirmed to be statistically significant by Kruskal-Wallis test (*p* < 0.0001).

Human chorionic gonadotropin beta subunit expression in various types of cancers is a commonly accepted fact [3,7,19]. Our previous studies also showed that nontrophoblastic gynecological tumors overexpress *CGB* genes [20–23].

High activity of *CGB3* and *CGB8* was previously reported to be linked with cancerogenesis [9,12] and synthesis of human chorionic gonadotropin beta subunit is now a recognised phenomenon in 30%–50% of malignant tumors [3,8,12,19]. In this study the expression of *CGB* coding genes was detected in all studied cases of ovarian cancer tissue. The previously published data, documenting CGB presence in about 50% of cancer patients’ serum and tumor tissues was obtained using immunohistochemistry, ELISA and conventional PCR, thus the detection limit of these study methods was lower compared to the qPCR technology used in the present study. Since our experimental design enables to detect even a very small number of *CGB* transcripts we were able to show CGB presence in all studied samples.

There is no definite explanation of total *CGB* and *CGB3*-*9* gene activity observed in ovarian tissue lacking cancerous changes. Sequence specific primers and hydrolysis probes used in qPCR study eliminate the possibility of false-positive results. This suggests that some cells in normal ovaries may have transcriptionally active *CGB* genes. The number of these cells may be very low, but the high sensitivity of qPCR enables their detection. Therefore, the level of gene expression, not the presence of genes’ transcripts should be verified in case of tumor cell detection.

### 2.2. *CGB1-2* Gene Activity Characterizes Ovarian Cancer

**Figure 3:**
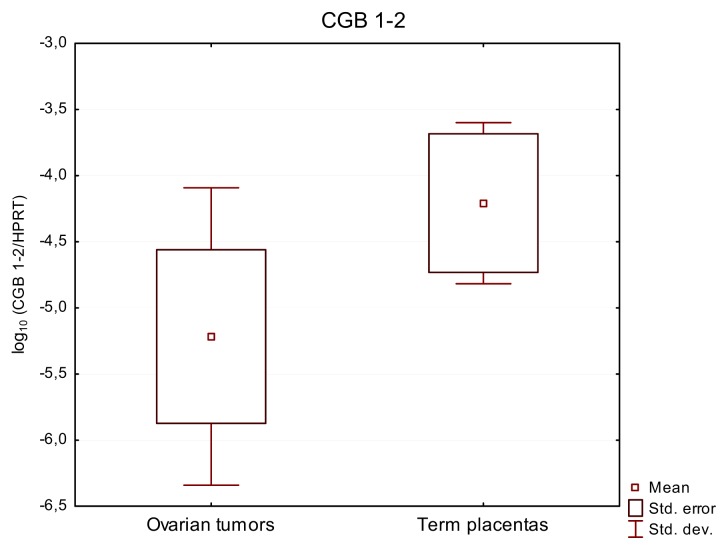
Relative expression of *CGB1-2* genes in ovarian carcinomas and term placentas. None of studied healthy ovaries exhibited expression of *CGB1-2*. Results are presented as the logarithm to the base 10.

In contrast to the expression of total *CGB* and *CGB3*-*9* found in all tumors, the activity of *CGB1-2* was observed in only 13 out of 32 (41%) studied cases of ovarian cancers. This group expressing *CGB1-2* included: 11 serous, 1 endometrioid and 1 clear cell carcinoma cases. 7 of these tumors were classified as G3 grade, 5 as G2 and 1 was of an undetermined grade. *CGB1-2* transcripts were detected in 6 out of 12 (50%) term placental samples (Figure 3, Table 1). Expression of these genes was not detected in any of the studied normal ovaries, lacking cancerous changes. Relative expression level of *CGB1-2* in all *CGB1-2* positive tumors and placentas was low (mean values: 7.27 × 10^−5^ and 1.84 × 10^−4^ respectively) and did not exceed 8.56 × 10^−4^.

Within the group of 13 samples of ovarian cancer expressing *CGB1* and *CGB2* genes a positive correlation between *CGB1-2* and *CGB3*-*9* activity was found (Spearman’s test *R* = 0.66, *p* < 0.05).

*CGB1* and *CGB2* expression in early placentas from both normal and complicated pregnancies as well as in some nontrophoblastic tissues was demonstrated before, however their expression was not tested in term placentas or gynecological cancer [24]. The highest *CGB1-2* gene activity was detected in normal intrauterine first trimester pregnancy placentas and ectopic pregnancy. Significant reduction of *CGB1-2* expression was noted in recurrent miscarriages [24], thus these gene expression activities may be a requirement for trophoblast invasion, immunological adaptation of the embryo and its further development.

The results of this study document, for the first time, the activity of *CGB1* and *CGB2* in ovarian cancer tissue. Until recently these genes were not examined very closely since they were regarded as pseudogenes. Recent studies demonstrated the presence of CGB genes’ transcripts in placenta, testes pituitary and in breast cancer tissue [10,14,16]. However, the contribution of *CGB1-2* to the summarized total CGB gene expression has been shown not to be proportional to their gene dosage [13]. The expression level of *CGB1-2* established for ovarian cancers evaluated in this study was also low, still its absence in normal ovaries and presence in ovarian cancer implies that this gene activity distinguishes cancer tissue.

**Figure 4:**
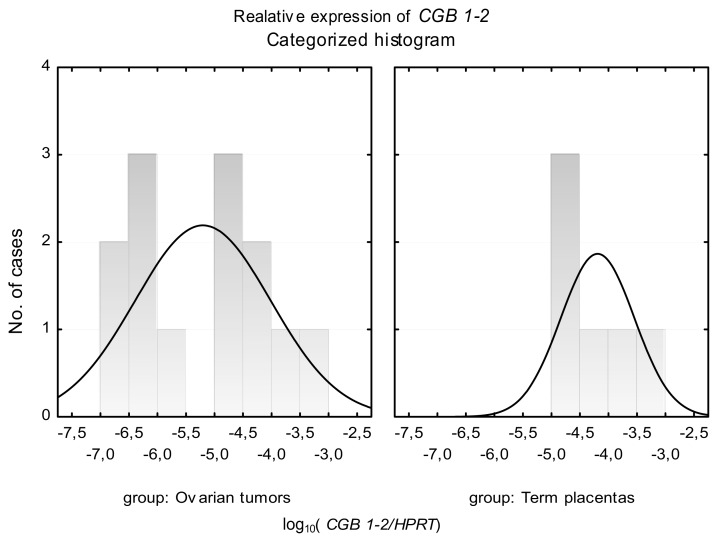
Categorized histogram of *CGB1-2* relative expression in tissues of normal ovaries, ovarian carcinomas and term placentas. Results are presented as the logarithm to the base 10. Data not normally distributed.

Analysis of *CGB1-2* expression in term placentas disclosed distribution of results in one coherent group. In the case of tumor tissues, gene activity was fitted into two distinct groups of distribution, as presented on the histogram (Figure 4). The first population showed lower expression of *CGB1-2* (mean value of *CGB1-2* relative expression 5.81 × 10^−7^), and the second demonstrated higher *CGB* activity (mean value of *CGB1-2* relative expression 1.35 × 10^−4^) (Figure 4).

There is no definite answer to the question why two distinct schemes of data distribution of results were observed. It can be postulated that the increased level of the *CGB1-2* gene expression could be associated with the differentiation stage of the tumor or degree of disease progression. However, increasing the number of patients is needed to prove this hypothesis.

Since the protein products of *CGB1* and *−2* genes have not yet been identified, their function and putative association with cancer remains unclear. In addition, several splicing variants of *CGB1-2* have been identified that seem to be expressed in a tissue specific manner [10,15,16,24]. Most of the variants are said to give rise to alternative protein products, however some authors claim that one of the *CGB2* variants can be translated to the common CGB protein [10,15]. The results obtained in this study assess total transcriptional activity of *CGB1* and *CGB2* genes in analyzed tissues. This was achieved by using primers amplifying all previously described *CGB1-2* splicing variants.

## 3. Experimental Section

### 3.1. Experimental Subjects

The study was approved by the ethics review board of Poznan University of Medical Sciences (Resolution No: 748/08) and all patients participated after informed consent. Samples of ovarian cancer were collected from 32 patients treated by surgery at the Department of Gynecologic Oncology, Poznan University of Medical Sciences in 2010–2012.

The histological subtypes of the ovarian carcinomas included: 25 serous, 3 endometrioid, 3 mucinous and 1 clear cell. Grading of the analyzed subtypes of ovarian carcinomas was as follows: G1—*n* = 3, G2—*n* = 12 and G3—*n* = 16 stage (Table 2). In one case the stage of carcinoma was not determined.

The control group included samples of ovaries (*n* = 9) that lacked cancerous changes as evaluated by a pathologist’s macroscopic and microscopic examination. The ovaries were obtained from postmenopausal patients who underwent total hysterectomy with additional oophorectomy due to myomas.

Fragments of placentas were collected from normal vaginal delivery at or over the 38th week of gestation (*n* = 12) at the Department of Perinatology of Poznan University of Medical Sciences, Poznan, Poland.

Tissue samples were stored in RNAlater buffer (Sigma Life Sciences, St. Louis, MO, USA) at −80 °C.

### 3.2. RNA Isolation

Total RNA was isolated from 100–300 mg of tissue homogenized in 1 mL of TriPure Isolation Reagent (Roche Diagnostics, Mannheim, Germany) according to manufacturer’s protocol. Air-dried pellet of RNA was resuspended in 50 μL of UltraPure DNase*/*RNase*-*Free Distilled Water (Invitrogen, Carlsbad, CA, USA). RNA quality and concentration was determined spectrophotometrically. RNA was stored at −80 °C prior to further steps.

### 3.3. cDNA Synthesis

One microgram of total RNA was used as template for reverse transcription using the oligo(dT)_10_ primer and Transcriptor Reverse Transcriptase (Roche Diagnostics, Mannheim, Germany) according to the manufacturer’s protocol.

### 3.4. qPCR

Assays were designed to enable analyses of genes encoding human chorionic beta subunit in three separate groups: *CGB1-2*, *CGB3-9* (*CGB3/9*, *CGB5*, *CGB6/7*, *CGB8*) and total *CGB* for collective quantification. Hydrolysis probes and primers used during reaction are presented in Table 3.

In order to analyze the expression level of *CGB1-2*, and *CGB3*-*9* qPCR using hydrolysis probes (Tib Molbiol, Berlin, Germany) was performed. Total *CGB* expression was assessed with SybrGreen also using qPCR. The relative expression of studied genes was normalized against *HPRT*. All primers were designed for exon-exon junction to exclude genomic DNA amplification. CGB1-2 primers allow analysis of all spliced variants, described previously [10,13,15].

The reaction mix for TaqMan reactions contained: 5 μL of cDNA; 1× TaqMan master mix (Roche Diagnostics, Mannheim, Germany) 0.1 μM hydrolysis probe (TaqMan); 0.4 μM of each primer. Hydrolysis probes for *CGB1-2* and *CGB3*-*9* were designed by Tib Molbiol. Phosphoribosylotransferase (*HPRT*) housekeeping gene assay was purchased from Universal Probe Library (Roche Diagnostics, Mannheim, Germany). qPCR program consisted of initial denaturation at 95 °C for 10 min followed by 45 3-step cycles: 95 °C/10 s hold for denaturation, 60 °C/30 s hold for primers and probes hybridization and product extension, and 72 °C/1 s hold for data acquisition.

SybrGreen qPCR reactions for total *CGB* and *HPRT* contained 3 μL of template cDNA, 1× SybrGreen master mix (Roche Diagnostics, Mannheim, Germany) and 0.4 μM of each primer. Reaction program consisted of initial denaturation at 95 °C for 10 min followed by 45 3-step cycles: 95 °C/10 s hold for denaturation, 54 °C/5 s hold for primers hybridization (63 °C/5 s in case of total *CGB*), 72 °C/8 s hold for product extension (72 °C/6 s in case of total *CGB*). The amplification process was followed by a melting curve acquisition.

PCR efficiencies were calculated from the standard curves, which were generated using serial dilutions of cDNA library obtained from testes and placenta. Relative expression of genes analyzed by TaqMan assays was normalized against *HPRT* (Human *HPRT* Gene Assay, Roche Diagnostics, Mannheim, Germany).

### 3.5. Statistical Analysis

All experiments were performed in triplicates, using independently synthesized cDNA. qPCR data was assembled using the LightCycler computer application software 4.05 dedicated for the LightCycler 2.0 (Roche Diagnostics, Mannheim, Germany). All data were analyzed using the Statistica 10 software package (StatSoft, Kraków, Poland).

Relative levels of total *CGB*, *CGB1-2* and *CGB3*-*9* expression in three studied groups: ovarian tumors, healthy ovaries and post-parturition placentas were correlated using Spearman’s test.

The differences between various groups were analyzed by the Kruskal-Wallis or student’s *t*-test. Tests were considered to be statistically significant if *p*-value was lower than 0.05.

## 4. Conclusions

The results of the study revealed that two genes encoding for human chorionic gonadotropin beta subunit: *CGB1* and *CGB2*, originally considered as pseudogenes, may be active during cancerogenesis. While *CGB1* and *CGB2* gene expression was not detected in normal ovary lacking cancerous changes, the presence of their transcripts characterised ovarian cancers. Thus the detection of human chorionic gonadotropin beta subunit genes *CGB1* and *CGB2* expression may indicate cancerogenous processes taking place in ovarian tissue.

## Figures and Tables

**Table 1:** *CGB* gene expression pattern within studied groups. For CGB total and CGB3-9 assays, median and extent of range (maximum divided by minimum) are presented. For CGB1-2 assay (data not normally distributed) mean and standard deviation are shown.

Group	*n*	CGB total			CGB3-9			CGB1-2		
		Number/% of positive samples	Median	Maximum/minimum	Number/% of positive samples	Median	Maximum/minimum	Number/% of positive samples	Mean	Standard deviation
Ovaries	9	9/100%	3.51 × 10^−3^	40X	9/100%	1.31 × 10^−5^	5X	0/0%	n/a	n/a
Ovarian cancer tissues	32	32/100%	6.48 × 10^−3^	5747X	32/100%	2.65 × 10^−4^	250896X	13/40%	7.27 × 10^−5^	0.0001722
Term placentas	12	12/100%	44.3	1164X	12/100%	22.1	271X	6/50%	1.84 × 10^−4^	0.0003322

**Table 2:** The histological subtype and grading of studied ovarian carcinomas.

Ovarian carcinomas by histotype and grade				

Tumor grade	Serous	Endometrioid	Mucinous	Clear cell
G1	1	0	2	0
G2	8	2	1	1
G3	15	1	0	0
not determined	1	0	0	0
total	25	3	3	1

**Table 3:** Primers and hydrolysis probes used in qPCR.

Gene	Hydrolysis probe 5′→3′	Sequence of primers 5′→3′		NCBI Reference Sequence
		Forward primer	Reverse primer	
***CGB 3-9***(1)	6FAM-ccgaggtytaaagccaggtacacsaggc-BBQ	gtgtcsagctcacyccagcatccta (2)	agcagcccctggaacatct	CGB 3 NM_000737.3 CGB 5 NM_033043.1 CGB 7 NM_033142.1 CGB 8 NM_033183.2
***CGB 1-2***(1)	6FAM-tcactccctgtctcactcccccacg-BBQ	ggtccgctgactcyggc (2)	cagcagcagcccctttgac	CGB 1 NM_033377.1 CGB 2 NM_033378.1
***CGB*_all**	n/a	tactgccccaccatgacc	cacggcgtaggagaccac	CGB 1 NM_033377.1 CGB 2 NM_033378.1 CGB 3 NM_000737.3 CGB 5 NM_033043.1 CGB 7 NM_033142.1 CGB 8 NM_033183.2
***HPRT*_Sybr**	n/a	tgaagagctattgtaatgaccagt	caaatccaacaaagtctggc	HPRT NM_0001942

(1) CGB3-9 and CGB1-2 hydrolysis probe were designed by Tib Molbiol;

(2) Code for degenerated base positions: S-G/C; Y-C/T.
